# BrEPS 2.0: Optimization of sequence pattern prediction for enzyme annotation

**DOI:** 10.1371/journal.pone.0182216

**Published:** 2017-07-27

**Authors:** Christian-Alexander Dudek, Henning Dannheim, Dietmar Schomburg

**Affiliations:** Department of Bioinformatics and Biochemistry, Braunschweig Integrated Centre of Systems Biology (BRICS), Technische Universität Braunschweig, 38106 Braunschweig, Germany; Universite Paris-Sud, FRANCE

## Abstract

The prediction of gene functions is crucial for a large number of different life science areas. Faster high throughput sequencing techniques generate more and larger datasets. The manual annotation by classical wet-lab experiments is not suitable for these large amounts of data. We showed earlier that the automatic sequence pattern-based BrEPS protocol, based on manually curated sequences, can be used for the prediction of enzymatic functions of genes. The growing sequence databases provide the opportunity for more reliable patterns, but are also a challenge for the implementation of automatic protocols. We reimplemented and optimized the BrEPS pattern generation to be applicable for larger datasets in an acceptable timescale. Primary improvement of the new BrEPS protocol is the enhanced data selection step. Manually curated annotations from Swiss-Prot are used as reliable source for function prediction of enzymes observed on protein level. The pool of sequences is extended by highly similar sequences from TrEMBL and SwissProt. This allows us to restrict the selection of Swiss-Prot entries, without losing the diversity of sequences needed to generate significant patterns. Additionally, a supporting pattern type was introduced by extending the patterns at semi-conserved positions with highly similar amino acids. Extended patterns have an increased complexity, increasing the chance to match more sequences, without losing the essential structural information of the pattern. To enhance the usability of the database, we introduced enzyme function prediction based on consensus EC numbers and IUBMB enzyme nomenclature. BrEPS is part of the Braunschweig Enzyme Database (BRENDA) and is available on a completely redesigned website and as download. The database can be downloaded and used with the BrEPScmd command line tool for large scale sequence analysis. The BrEPS website and downloads for the database creation tool, command line tool and database are freely accessible at http://breps.tu-bs.de.

## Introduction

In the last decades, a large number of full genome sequencing projects have generated a huge amount of genomic data, but the function of most of the gene products is still unclear. Experimental annotation and functional study of all uncharacterized proteins is not possible at the speed the data grows [1]. Most of the methods for protein function prediction are based on sequence homology. It is commonly accepted that a high sequence similarity between two sequences indicates similar function. Different approaches using sequence homology have evolved to propose functions of previously uncharacterized proteins, based on proteins with experimentally determined functions. But with decreasing sequence similarity, a functional annotation is not always suitable [2]. For distantly related proteins, a simple homology-based function prediction often yields a highly questionable annotation [3]. But also isofunctional enzymes with low similarity often share essential amino acid positions, for example in the catalytic center or residues essential for folding [2]. One approach, which makes use of this fact is to compute sequence patterns form existing, well annotated sequences. Relationships between functional related sequences or domains can be described using clusters of specific amino acid residues. Those patterns—also named profiles, fingerprints or signatures depending on the publication [3] and method of generation—are specific arrangements of amino acids that are characteristic for a protein. For example, amino acid sequences that are known to form secondary structures like sheets, helices or bridges can be described with patterns [4]. Different approaches and aims have been evolved over time to provide pattern based genome annotation. ProDom [5] and Pfam [6] provide patterns related to protein domains while other databases focus on patterns to categorize proteins into families, like PRINTS [7], HAMAP [8] and PRIAM [9]. InterPro is a database classifying protein sequences into families predicting important domains and sites [10]. InterPro incorporates 14 different databases, including those mentioned above and other databases like TIGRFAMs [11], CCD [12] and SFLD [13]. The Braunschweig Enzyme Pattern Search (BrEPS) is a fully automated protocol, that generates enzyme specific sequence patterns based on manually annotated Swiss-Prot sequences [14].

Here we present BrEPS 2.0, the reimplementation of the original BrEPS protocol, enhanced with a more reliable data-source, an additional pattern type and more refinements to optimize the pattern creation. Additionally, we implemented a state-of-the-art, new web-service to search the BrEPS database and a simple but powerful command line program for batch sequence analysis.

## Methods

### Implementation

**Fig 1:**
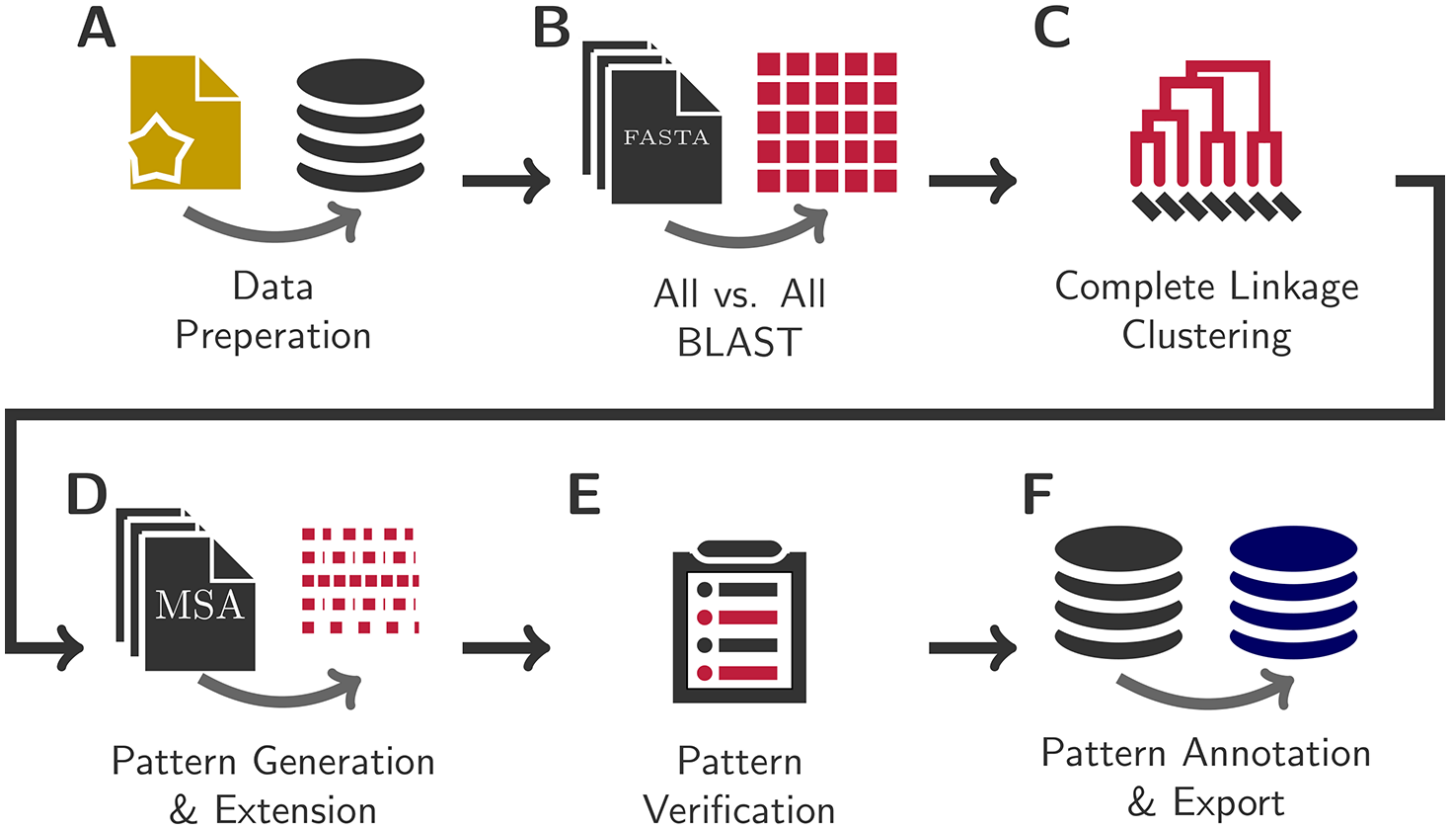
The BrEPS 2.0 workflow. The protocol consists of six steps to generate the BrEPS database. A: Selection and preparation of sequences. B: All-vs-all BLAST of sequences. C: Complete linkage clustering based on the E-value from BLAST. D: Multiple sequence alignment and pattern creation on selected nodes. E: Pattern verification. F: Preparation of the final database.

We reimplemented the original BrEPS protocol to be able to process larger datasets in an acceptable time frame. The original BrEPS workflow [14] was used as guide for the new implementation. BrEPS 2.0 consists of six steps (Fig 1). The first step is the selection of sequence data from the UniProt Knowledgebase [15] (Fig 1A). In the second step, all enzyme sequences are aligned to each other, using NCBIs BLAST+ [16] program (Fig 1B). The third step is the agglomerative hierarchical clustering of sequences, using the E-value obtained from the BLAST run, using the complete linkage clustering algorithm [17, 18] (Fig 1C). In the fourth step, a multiple sequence alignments (MSA) are performed on certain cluster nodes, using Clustal Omega [19]. Based on the MSA consensus lines, the BrEPS patterns are generated (Fig 1D). All patterns get verified in the fifth step. The prediction quality of the patterns is verified by comparison with the initial database and non-enzymes. The positive predictive value is calculated based on matches of EC numbers (Fig 1E). A final step was introduced in BrEPS 2.0, that creates the final compressed BrEPS database (Fig 1F). The BrEPS 2.0 protocol follows the rules of the original protocol, major optimizations in the new protocol are described in more detail further below.

### Data selection

The most significant change in the optimized BrEPS protocol is the selection of data used for pattern generation. The original protocol relies on enzyme sequences and annotations from Swiss-Prot, the manually annotated subset of UniPotKB. Entries with certain keywords (putative, hypothetical, fragment, probable, possible and potential) are omitted. Also, the sequences need to have a minimum length of 100 amino acids [14]. BrEPS uses parsed UniProt enzyme data, selected with different filters. All used filters are described in Table 1

**Table 1:** Filters applied to UniProt protein entries to parse enzyme data from UniProt flatfiles.

Filter	Field	Value	BrEPS	BrEPS 2.0
Length	SQ	100-7000 aa	yes	yes
Keywords	DE	not putative, hypothetical, fragment, probable, possible and potential	yes	yes
EC number	DE	present	yes	yes
Evidence	PE	1	no	yes
Publication*	RX	present	no	yes

*: Only for additional sequences from TrEMBL.

**Fig 2:**
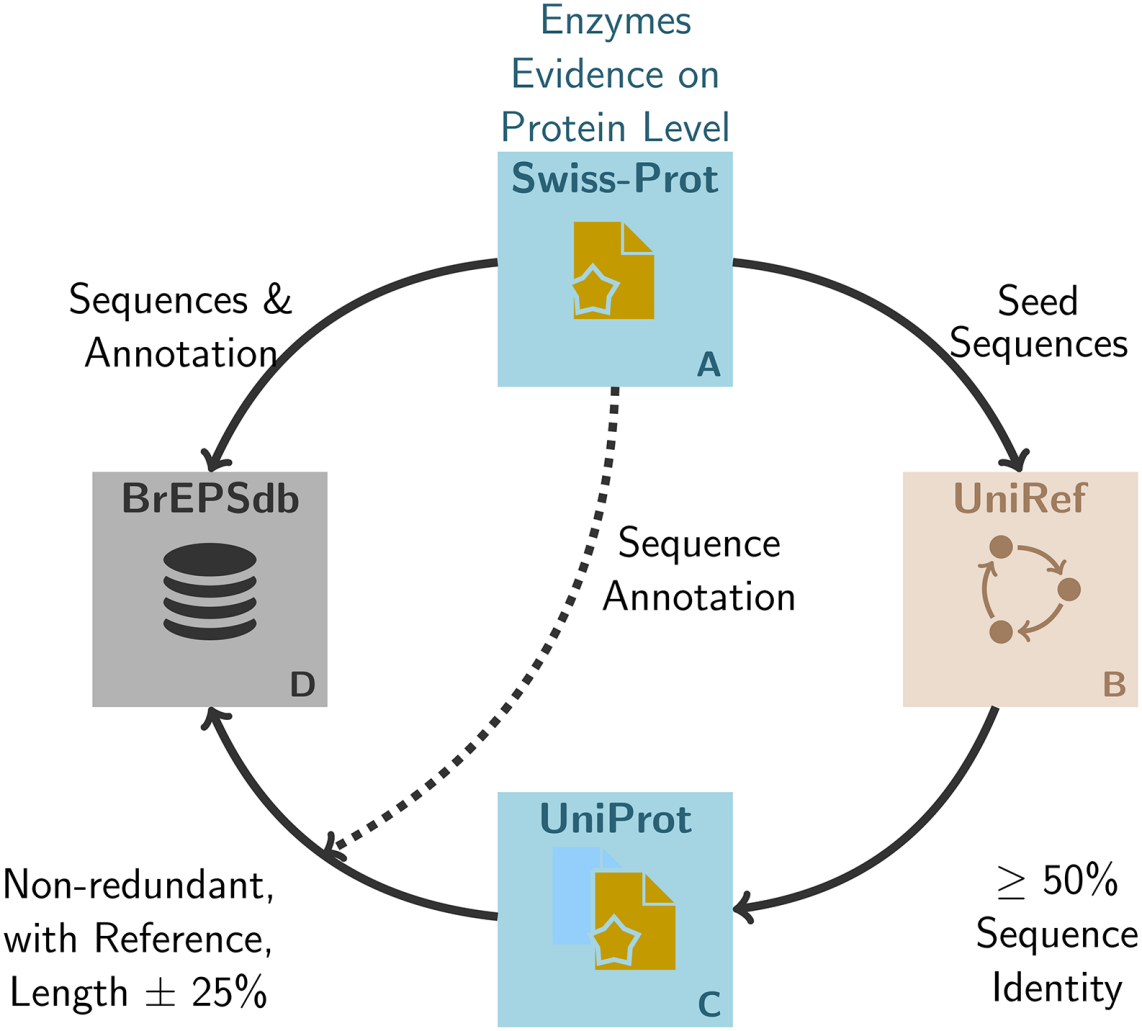
Detailed overview of the new data selection. Only Swiss-Prot sequences with evidence on protein level (A) are used as seed sequences to retrieve additional, non-redundant sequences from TrEMBL and Swiss-Prot using UniRef references with >= 50% sequence identity (B and C). These additional sequences get the corresponding Swiss-Prot annotation and are merged with the seed sequences into one database (D).

To further enhance the quality of selected sequences, the new protocol additionally uses only Swiss-Prot sequences with evidence on protein level (1 in the PE field). Using this qualifier, we only select sequences of proteins, that have been observed experimentally, to avoid the inclusion of sequences derived from predicted gene translations [20]. As most Swiss-Prot annotations are derived from homology, less sequences are selected from the new implementation compared to the original data selection. To generate a larger pool of sequences, we implemented the protocol shown in Fig 2. We use the Swiss-Prot sequences as seed sequences (Fig 2A) to retrieve additional sequences from TrEMBL and Swiss-Prot. To select appropriate sequences, UniProt Reference Clusters (UniRef [21]) are used to find sequences with sequence identity of 50% or greater for every seed sequence (Fig 2B). Similar sequences are selected, if length deviation of the sequence to the seed sequence is not larger or smaller than 25%. To archive the highest possible quality for the additional sequences we only select entries which have a publication (cross reference, RX field) annotated (Fig 2C). All additional entries get the annotation of the corresponding Swiss-Prot seed entry to apply the high quality annotation to the additional sequences. Finally, all seed sequences and the similar sequences are merged together into the working database (Fig 2D).

Using this method, the initial Swiss-Prot seed sequences are enriched with additional non-redundant sequences for the pattern generation. This increases the number of sequences for the pattern generation. For the final verification, non-enzyme sequences with evidence on protein level are stored as well.

### Pattern extension

BrEPS pattern creation is based on multiple sequence alignment (MSA) output. For the determination the essential amino acids for an enzyme, both the number of sequences and the inclusion of sequences with a larger evolutionary distance is crucial. Therefore, a high number of sequences is needed to generate significant patterns. As this is not given for all enzymes available, we implemented a method to extend BrEPS patterns to a certain degree, by adding similar amino acids to semi-conserved amino acid positions, consisting of multiple highly similar amino acids. In Clustal Omega, PAM250 [22] is used to determine the similarity of amino acids. Based on the PAM250 matrix, we determined the amino acids more similar to all pairs of amino acids with a PAM score greater than 0.5. An additional amino acid is added if the amino acid is at least as similar to the amino acid pair as the amino acids in the alignment. This procedure leads to sets of amino acids that can complement a semi-conserved positions with other highly similar amino acids (Table 2).

**Table 2:** Similarity sets created for every amino acid pair to complement semi-conserved amino acid positions with other highly similar amino acids.

Pair	Score	Similarity set	Pair	Score	Similarity set
EN	0.9	D	KQ	1.5	R
EK	1.2	Q	QR	1.5	K
MV	1.6	IL	IM	2.5	L
HR	0.6	KQ	AT	0.6	S
FW	3.6	Y	FI	1.0	LM
KN	0.8	E	NQ	0.7	DEHK
LV	1.8	I	FM	1.6	L
HK	0.6	NQR	DQ	0.9	E

The scores are derived from Gonnets PAM250 matrix [22].

**Fig 3:**
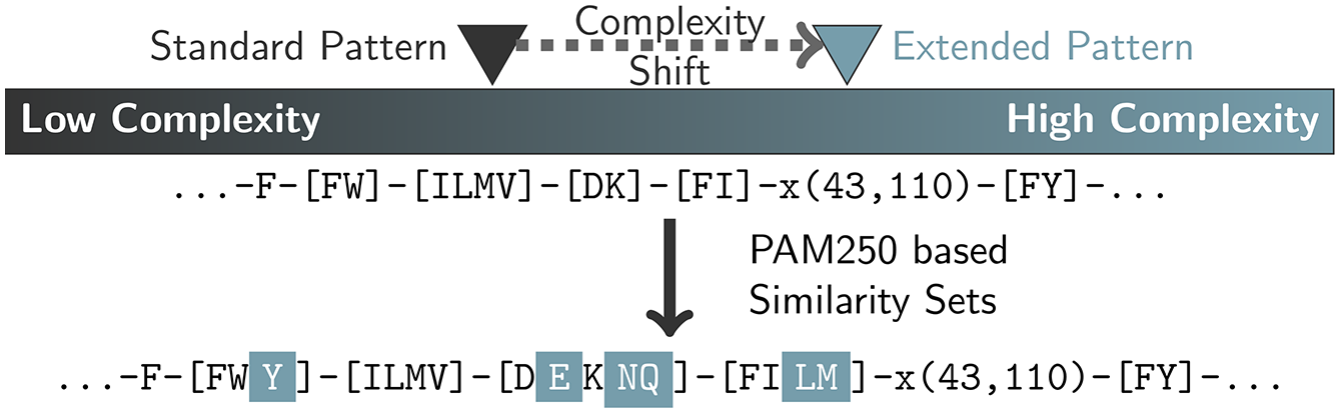
Extended patterns example. Semi-conserved pattern positions are extended with amino acids from PAM250-based similarity sets.

Amino acids are added to semi-conserved positions with two or more amino acids, using the corresponding similarity set, leading to a shift in pattern complexity. For semi-conserved positions with more than two amino acids, the similarity sets for all amino acid combinations are combined (Fig 3). The extended patterns are saved in addition to the patterns without amino acid extension (standard patterns). This way, extended patterns have a higher complexity compared to standrad patterns. To ensure, that the extended patterns meet the high quality of the standard patterns, all extended patterns are verified using the same procedure as used for the standard patterns.

### Pattern annotation

The generated BrEPS patterns are annotated using the EC number of the corresponding sequences they were created from, which represents the enzymatic function. These patterns are not always generated from sequences with the same EC number. To simplify the BrEPS search results, we propose a consensus EC number, which is based on all EC numbers of all sequences used for pattern generation. A partial EC number is created based on consensus positions in the EC number. For example, the EC numbers 1.1.1.1 and 1.1.2.1 would give the consensus EC number 1.1.-.-. If none of the four digits match, all EC numbers will be proposed as possible functions. For multi-functional enzymes with different annotated EC numbers, we assume that distinct domains are responsible for the different catalytic functions. In this case, the different EC numbers (or parts) are only proposed if they occur in all sequences. Further examples for the consensus EC number creation are shown in Table 3.

**Table 3:** Proposed EC number examples for patterns created from sequences with different EC numbers.

#	EC numbers	Proposed EC	Reason
1	1.1.2.1	1.1.-.-	Consensus of first two digits
2	1.1.1.1		
1	2.1.3.1	2.1.3.1 OR 6.4.1.1	No consensus, both functions are possible
2	6.4.1.1		
1	6.4.1.2 &	6.4.1.2 AND 6.3.4.14	Two functions for one enzyme
	6.3.4.14		
1	6.4.1.7	6.4.1.-	Consensus in only one EC number
2	6.4.1.2 &		
	6.3.4.14		

Multiple EC numbers for one enzyme are separated with “&”. Consensus parts of EC numbers are underlined.

Based on the consensus EC number, the function is annotated from the IUBMB database [23]. The recommended enzyme name is used for full EC numbers. For partial EC numbers the class, subclass and sub-subclass (if given) description is used instead. All EC numbers are stored with the number of occurrences, to keep track of the composition of the proposed EC number. Also, the UniProt accessions are kept for reference.

### Minor changes

Many changes have been made while reimplementing BrEPS. We also incorporated the latest versions of BLAST+ and Clustal Omega into the BrEPS pipeline. Below we discuss some minor refinements we implemented to further enhance pattern quality or speed and stability of the program.

#### BLAST

Highly similar sequences get a low E-value as a BLAST result. This can be problematic, because E-values smaller than 1e-180 are returned as zero. In the clustering step, those highly similar sequences are randomly clustered together. To archive better reproducibility, E-values of zero are substituted using the BLAST score to allow differentiation of those sequences. The substitution of the E-value *E* with the score *s* is defined as *E* = 10^−180^ ⋅ 10^−*s*^. This refinement adds a higher resolution for the clustering of highly similar sequences.

#### Clustering

A major bottleneck in the original BrEPS pipeline was the clustering step, because it needed an extensive amount of main memory for the clustering. We implemented a database assisted clustering algorithm based on the optimally efficient clink algorithm [17]. The distance matrix is saved and modified in the MySQL database and only dictionaries for nearest neighbors and their distances are stored in the main memory. Using this algorithm, the clustering needs a negligible amount of main memory, while running in an acceptable timescale of less than a week on a single CPU core.

#### Verification

The verification step is crucial to ensure a high quality of the generated patterns. To use a reliable source of sequences for verification, all patterns are verified, using the Swiss-Prot seed sequences and non-enzymes with evidence on protein level. The original protocol used a positive predictive value (PPV, Eq (2)) cut-off of 0.75 for the exclusion of patterns. For BrEPS 2.0 patterns, only 0.6% of the generated patterns are below the 0.75 PPV threshold. To increase pattern quality, we raised the PPV cut-off to 1.0 and exclude all patterns that produce false positive hits. Using this cut-off, 3.0% of the patterns are excluded.

### Runtime and resource usage

BrEPS 2.0 was implemented using Python 2.7, with MySQL as database backend. All steps, except the clustering step, are parallelized for usage on a computer cluster. On a computer cluster with 36 CPU cores, BrEPS 2.0 takes about 10 days to create and verify the patterns. The maximum usage of main memory is about 1-2 GB (pattern verification step).

## Results and discussion

### Data selection

The new data selection creates a set of sequences for pattern generation. The working database for the UniProt release 2017_1 contained 29,765 enzyme sequences with evidence on protein level. In comparison, the data selection of BrEPS 1 would have selected 197,451 SwissProt sequences. The 30,317 seed sequences were used to select 348,603 similar non-redundant sequences from TrEMBL. For the verification of the generated patterns, 196,554 non-enzyme sequences were parsed from UniProt.

### Validation of extended patterns

The purpose of the inclusion of extended patterns is an increase of the prediction rate for BrEPS patterns. This increase should be visible when a sequence analysis is performed with both pattern types and compare the positive and negative hits for each pattern. The analysis showed that the majority of patterns have an equal amount of true positive and false positive hits. This fact is not surprising, because the verification uses the original set of sequences used for pattern creation and the standard patterns should hit the sequences they were created from. 104 extended patterns created additional positive hits, which means, that the extended pattern matched a sequence with the correct EC number which was not matched by the standard pattern. On the other hand, only 23 extended patterns produced more false positive hits than the corresponding standard pattern.

The analysis showed, that the use of extended patterns increases the detection rate. One should keep in mind, that the higher complexity of extended patterns may lead to false positive hits. But we minimize this risk by excluding all patterns creating false positive hits during the verification step.

### Evaluation of pattern quality

To evaluate the prediction rate of the patterns generated with the new BrEPS protocol, a standard of truth independent from the dataset used for pattern creation has to be defined. Here, a reliable source of sequences in combination with an annotated EC number is needed to check for the prediction rate of patterns. The enzyme database BRENDA [24] matches the criteria as reliable source of sequences with annotated EC numbers. BRENDA gets the sequences from UniProt, but does their own annotation using literature data of enzymes with experimental verification. Therefore, the annotation for the sequences in BRENDA is independent from and superior to UniProt, as Swiss-Prot entries with evidence on protein level does not strictly include experimental evidence of the annotated function. BRENDA contains sequences not just from Swiss-Prot, but also from TrEMBL. To get a solid set of sequences for the evaluation, all BRENDA sequences with an annotated EC number were used. The sequence set for evaluation consisted of 14,106 Swiss-Prot sequences and 11,384 TrEMBL sequences.

#### Evaluation procedure

The evaluation set of sequences was matched against the BrEPS database using the BrEPScmd command line tool. Every pattern can either hit or miss a sequence. If a sequence is not found by any pattern in the BrEPS database, the sequence was considered as a negative (N) match. Positive matches were further classified by comparison of the EC numbers of the BRENDA annotation and the BrEPS annotation. Here, two levels of EC number matches were used:

**Strict match**: The EC number of the sequence exactly matches one of the BrEPS EC numbers, incomplete EC numbers (i.e. 1.1.1.-) do not match.

**Fuzzy match**: Like the strict match, but incomplete EC numbers can match (i.e. 1.1.1.- can match 1.1.1.1). Undefined positions count as wild card character.

Hits were counted as true positive (TP), if one of the pattern EC numbers matched the EC number annotated in BRENDA, otherwise as false positive (FP) hit. As the sequences are derived from UniProt, it is possible that it was used for pattern generation, therefor we checked if the sequence were present in the BrEPS working database to assess the reliability of the evaluation.

$$\begin{array}{ccc} DR & = & \frac{TP+FP}{TP+FP+N} \end{array}$$ (1)

$$\begin{array}{ccc} PPV & = & \frac{TP}{TP+FP} \end{array}$$ (2)

#### Evaluation results

For the comparison with the original BrEPS implementation, we used the UniProt release 2014_10 with the new protocol to generate BrEPS patterns. We used the described evaluation procedure with the original BrEPS patterns (BrEPS 1.0) and our reimplementation with the described enhancements (BrEPS 2.0). The evaluation results (Table 4) show an increased detection rate (Eq (1)) for BrEPS 2.0 compared with BrEPS 1.0 of more than 50%. The positive predictive value (PPV, Eq (2)) of BrEPS varies, depending on the EC number matching level used. For the strict matching, BrEPS 2.0 has a higher PPV than BrEPS 1.0, while for the fuzzy matching, both protocols are on par with about 90% PPV. The extended patterns of BrEPS 2.0 contributed to the fuzzy matches with 101 true positive hits and 29 false positive hits which had not been hit by a standard pattern.

**Table 4:** Evaluation results for BrEPS 1.0 and BrEPS 2.0 using UniProt release 2014_10.

**BrEPS 1.0**					
	TP	FP	N	PPV [%]	DR [%]
strict	8840	1711	13426	83.78	44.00
fuzzy	9514	1037	13426	90.17	44.00
in BrEPS	5674	1107	0	83.67	100.00
not in BrEPS	3166	604	13426	83.98	21.92
**BrEPS 2.0**					
	TP	FP	N	PPV [%]	DR [%]
strict	11126	1798	11053	86.09	53.90
fuzzy	11673	1251	11053	90.32	53.90
extended	101	29	0	77.69	100.00
in BrEPS	6766	1109	0	85.92	100.00
not in BrEPS	4360	689	11053	86.35	31.36

The sequences used for evaluation from BRENDA are also part of the UniProt database, and therefore, could have been part of the BrEPS pattern generation process. BrEPS 2.0 was able to detect more sequences which have not been part of the pattern generation with a positive predictive value of 86.35% and a detection rate of 31.36%. It should be noted, that the presence of a sequence in the working database does not imply, that a pattern is associated with it. However, due to the addition of similar TrEMBL and Swiss-Prot sequences, it is possible that a similar sequence is present. We also created the BrEPS database using UniProt release 2017_01 (BrEPS version 2017.1) and evaluated the patterns (Table 5). Due to the larger UniProt database, the BrEPS patterns have a higher positive predictive value compared to the BrEPS pattern based on UniProt release 2014_10. This shows, that future updates will further enhance BrEPS patterns due to UniProt data updates.

**Table 5:** Evaluation results for BrEPS 2.0 using UniProt release 2017_01.

	TP	FP	N	PPV [%]	DR [%]
strict	11951	1740	10286	87.29	57.10
fuzzy	12444	1247	10286	90.89	57.10
extended	83	19	0	81.37	100.00
in BrEPS	7398	1097	0	87.09	100.00
not in BrEPS	4553	643	10286	87.63	33.56

In summary, BrEPS 2.0 patterns have a higher detection rate and positive predictive value for the strict EC number matching, compared with the original protocol. This increase is also applied for sequences not used for pattern generation. It could also be shown, that the extended patterns can positively contribute to the pool of BrEPS patterns.

### Comparison with InterPro

InterPro is a manually annotated database, that incorporates different databases and approaches to generate a comprehensive resource for annotation of protein functions [10]. We used our standard of truth to evaluate InterPro version 63.0 data, using a comparable approach to the BrEPS evaluation. InterPro provides different classifications, so multiple and contrary annotations are possible for a protein. Therefore, we used a strict and a loose evaluation. For the strict evaluation all EC numbers of the InterPro results have to match the standard of truth EC number to generate a true positive hit, otherwise a false positive hit is generated. For the loose evaluation it is sufficient that only one InterPro results matches the standard of truth EC number. If no EC number is annotated for an InterPro result a negative hit is generated.

The results of our analysis are shown in Table 6. We compared the evaluation of the strict matching of BrEPS 2.0 (as described above) with the two InterPro evaluations. The detection rate of InterPro (48.30%) is lower than the BrEPS 2.0 detection rate. The positive predictive value for the strict evaluation is also lower to the BrEPS evaluation. However, the loose evaluation is almost on par with the BrEPS PPV.

**Table 6:** Comparison of InterPro 63.0 with BrEPS 2017.1.

	TP	FP	N	PPV [%]	DR [%]
InterPro (strict)	9459	2121	12397	81.68	48.30
InterPro (loose)	9965	1615	12397	86.05	48.30
BrEPS 2.0 (strict)	11951	1740	10286	87.29	57.10

The new BrEPS protocol performs significantly better than InterPro in the prediction of enzymatic functions. However, the main focus of InterPro lies on classification into families and domains. Therefore, BrEPS and InterPro do not directly compete for the functional annotation. The main aim of InterPro is the detection of protein families and functional domains, whereas BrEPS aims for enzyme functions. Only 13.65% of the InterPro entries had an annotated EC number cross-references.

## Conclusion

We have completely reimplemented the original BrEPS protocol for generation of enzyme specific sequence patterns. The new protocol has a high maintainability for future usage of the pattern generation pipeline. The new data selection criteria ensures a high-quality sequence source for pattern generation by using enzyme sequences with evidence on protein level. The newly introduced extended patterns are based on the amino acid similarity commonly found in proteins. While retaining the initial structure of the pattern, the extension with similar amino acids create slightly more complex patterns. Analysis of the BrEPS verification step and the evaluation with BRENDA annotations showed, that the enhanced complexity of the extended patterns is detectable and can improve the pool of BrEPS patterns.

The evaluation was performed using a non-restricted set of sequences based on BRENDA annotations. It could be shown, that the BrEPS 2.0 patterns have a high detection rate and positive predictive value, with the limitation for similar sequences used for generation. The new protocol shows improved values for the detection rate and positive predictive value, although the initial pool of Swiss-Prot sequences and annotations was reduced. However, as all similarity based methods, BrEPS is limited to enzymes which are well described and characterized. Keeping the limitations in mind, BrEPS is a highly sensitive source for enzyme annotation and especially, due to the high sensitivity, for the verification of annotations. Additionally, regular updates of the BrEPS database will further improve the detection rate, as long as the Swiss-Prot part of UniProt grows (compare Tables 4 and 5).

We showed that BrEPS 2.0 can compete with InterPro, a database based on different approaches and databases to classify proteins into families and detect functional domains. However, BrEPS and InterPro aim for distinct purposes in the field of enzyme function predictions.

## Availability

The BrEPS database is freely available using the new designed web-service at http://breps.tu-bs.de. The web-service features a BrEPS sequence search and a full-text search for patterns, as well as a comprehensive overview for all patterns with full annotation of EC numbers and accessions. Additionally, the BrEPS database can be downloaded in MySQL and SQLite format. For large scale batch analysis, we offer a command line tool BrEPScmd, written in Python, which can be used to search the downloadable database. The database and command line tool (BrEPScmd) are available at our website http://breps.tu-bs.de. The BrEPS source-code is available under GPL-3.0 license on our website.

## Supporting information

S1 Table Evaluation results of BrEPS 1.0 patterns with the BRENDA standard of truth. (ODS)

S2 Table Evaluation results of BrEPS 2.0 patterns (from UniProt release 2014_10) with the BRENDA standard of truth. (ODS)

S3 Table Evaluation results of BrEPS 2.0 patterns (from UniProt release 2017_01) with the BRENDA standard of truth. (ODS)

S4 Table Evaluation results of InterPro version 63.0 with the BRENDA standard of truth. (ODS)
